# No evidence of *Wuchereria bancrofti* infection in *Anopheles* species after 10 years without mass drug administration: a molecular xenomonitoring study in Hauts-Bassins, Burkina Faso

**DOI:** 10.1186/s41182-025-00808-3

**Published:** 2025-09-25

**Authors:** Achille Sindimbasba Nikièma, Lassane Koala, Rabila Bamogo, Simon P. Sawadogo, Arielle Bettina Sandra Badiel, Ali Ouari, Abdoul-Aziz Millogo, Clarisse Bougouma, Mamadou Sermé, Soungalo Traoré, Babacar Faye, Roch Kounbobr Dabiré

**Affiliations:** 1 Institut de Recherche en Sciences de la Santé, Direction Régionale de l’Ouest, Ministère de l’Enseignement Supérieur de la Recherche et de l’Innovation, Bobo-Dioulasso, Burkina Faso; 2 Université Cheikh Anta Diop, Dakar, Sénégal; 3 Programme National de lutte contre les Maladies Tropicales Négligées, Ministère de la Santé, Ouagadougou, Burkina Faso

**Keywords:** Lymphatic Filariasis, Molecular Xenomonitoring, *An. gambiae s.l*., *An. funestus*, *An. nili*, *Wuchereria bancrofti*, Implementation Unit, Burkina Faso

## Abstract

**Background:**

The Lymphatic Filariasis Elimination Programme was launched in Burkina Faso in 2001 aiming to eliminate the disease as a public health concern through mass drug administration (MDA). After eight years of MDA, the Hauts-Bassins region successfully passed the Transmission Assessment Survey (TAS), which led to the MDA being stopped. This study aims to assess whether parasite transmission has resurfaced in areas where MDA was stopped more than ten years ago.

**Methods:**

A cross-sectional entomological survey was conducted in the villages of Tiebalogo and Tondogosso, in the Hauts-Bassins region. From August to December 2022, adult mosquitoes were collected using Human Landing Collection (HLC) indoor and outdoor, Window Exit Trap (WET) and Pyrethrum Spray Collection (PSC). Mosquitoes were identified morphologically. Genomic DNAs extracted from *An. gambiae s.l., An. funestus, An. nili* were amplified by PCR for *Wuchereria bancrofti* parasite detection.

**Results:**

A total of 2688 mosquitoes were collected in both study sites, with 630 being collected in Tondogosso and 2058 in Tiebalogo. The *An. gambiae s.l.* was the predominant mosquitoes, with high numbers being collected in both sites. Of those collected in Tiebalogo, 1786 (86.78%) were identified as *An. gambiae s.l.,* while 373 (59.21%) were identified in Tondogosso. The HLC method collected the greatest number of mosquitoes, followed by the PSC and WET methods. No *Wuchereria bancrofti* DNA was detected in any of the mosquito pools analyzed in both sites.

**Conclusions:**

These findings provide further evidence that there is no Lymphatic Filariasis transmission occurring in Hauts-Bassin’s post-TAS area. Molecular xenomonitoring of the filarial parasite which is a sensitive tool, could also serve as a complementary tool for monitoring transmission in post-MDA area and help national neglected tropical disease control program with surveillance in these areas.

## Background

Lymphatic filariasis (LF) is an infectious disease spread by mosquitoes and caused by filarial worms such as *Wuchereria bancrofti, Brugia malayi*, and *B. timori*. In Africa, where more than 90% of infected people live, LF is caused by *Wuchereria bancrofti*. In West African rural areas, *Anopheles* mosquitoes are the most responsible of parasite transmission with *An. gambiae s.l.* being the major vector [1]*.*

In 2000, the World Health Organization (WHO) launched the Global Programme to Eliminate Lymphatic Filariasis (GPELF) with the goal to eliminate the disease as a public health problem by 2020 [2]. At that time the goal was reviewed and new objectives were set for the year 2030 [3]. The main strategy established to interrupt transmission was mass administration of anthelminthics to entire at-risk populations [4, 5]. Since the implementation of the MDA, significant progress has been made, including a 74% reduction in global infection by 2020. By 2023, the WHO had validated that twenty-one countries and territories had eliminated lymphatic filariasis as a public health problem [6]**.** To achieve the goals outlined in the 2030 Roadmap, new diagnostic tools and strategies are necessary to monitor and evaluate progress during the post-MDA phase. To this end, molecular xenomonitoring (MX), a more sensible tool could complement in post-TAS efforts [7].

Burkina Faso was one of the first countries in sub-Saharan Africa to initiate a mass drug administration (MDA) program using Ivermectin and Albendazole to eliminate LF as a public health problem. The MDA was firstly conducted in 2001, initially in the most endemic districts, and then progressively expanded to cover all endemic districts by 2006 [8]. In the Hauts-Bassins region, the MDA was initiated in 2003 in two implementation evaluation units. After six years of the MDA, with over 65% coverage achieved, this region successfully completed the TAS and the MDA was stopped in 2011 [9]. The TAS is the only approach recommended by the WHO for stopping or continuing MDA in an implementation unit. MDA is stopped in implementation units if the prevalence of microfilaremia is less than 1% or the prevalence of antigenemia is less than 2% [10]. Thus, it is expected that transmission will be naturally cease in the years following the cessation of MDA, as the infection is present at low levels in the human population and vectors can no longer facilitate transmission [11]. However, the risk of lymphatic filariasis transmission resurging after stopping MDA based only on TAS is unknown [12]. Previous studies have reported ongoing transmission in countries that stopped MDA after successfully passing TAS [7, 13–15]. These situations have showed that TAS may be inadequate for making decisions to stop MDA, in areas with low prevalence.

MX is a non-invasive, indirect method of detecting the genetic material of LF parasites in humans by analyzing mosquito vectors. Although this method is sensitive, it is still being standardized for the purpose of lymphatic filariasis surveillance [16]. Until the molecular xenomonitoring protocol is standardized, the current protocol could provide guidance on the presence of *Wuchereria bancrofti* in a human population in a post-MDA area at a given point in time. This enables national programs for lymphatic filariasis elimination in endemic countries to implement corrective measures before the infection spreads in areas that have stopped MDA.

Since 2011, the implementation units in the Hauts-Bassins region of Burkina Faso, have successfully passed TAS, with antigenemia levels below 2% leading to the attainment of the goals of the MDA program. Therefore, it is essential to verify that regions that have ceased MDA have not experienced a resurgence of infection while awaiting the completion of TAS in all remaining implementation units and the subsequent submission of the country’s dossier to the WHO for the validation of LF elimination. This study, which employed a MX approach, to determine if LF transmission has resurfaced in implementation units in the Hauts-Bassins region, where MDA ceased about ten years. This study is expected to provide evidence towards LF elimination and inform the development of an entomological protocol for post-MDA surveillance of LF in the remaining post-MDA implementation units of Burkina Faso.

## Methods

### Study area and selection of study sites

The study was carried out in the Hauts-Bassins region located in the western part of Burkina Faso. The region’s climate is of Sudanian type with annual rainfall varying between 900 and 1100 mm. The region experiences two distinct seasons: a dry season and a rainy season. The population of the region is estimated at 2,349,820 inhabitants [17].

**Fig. 1:**
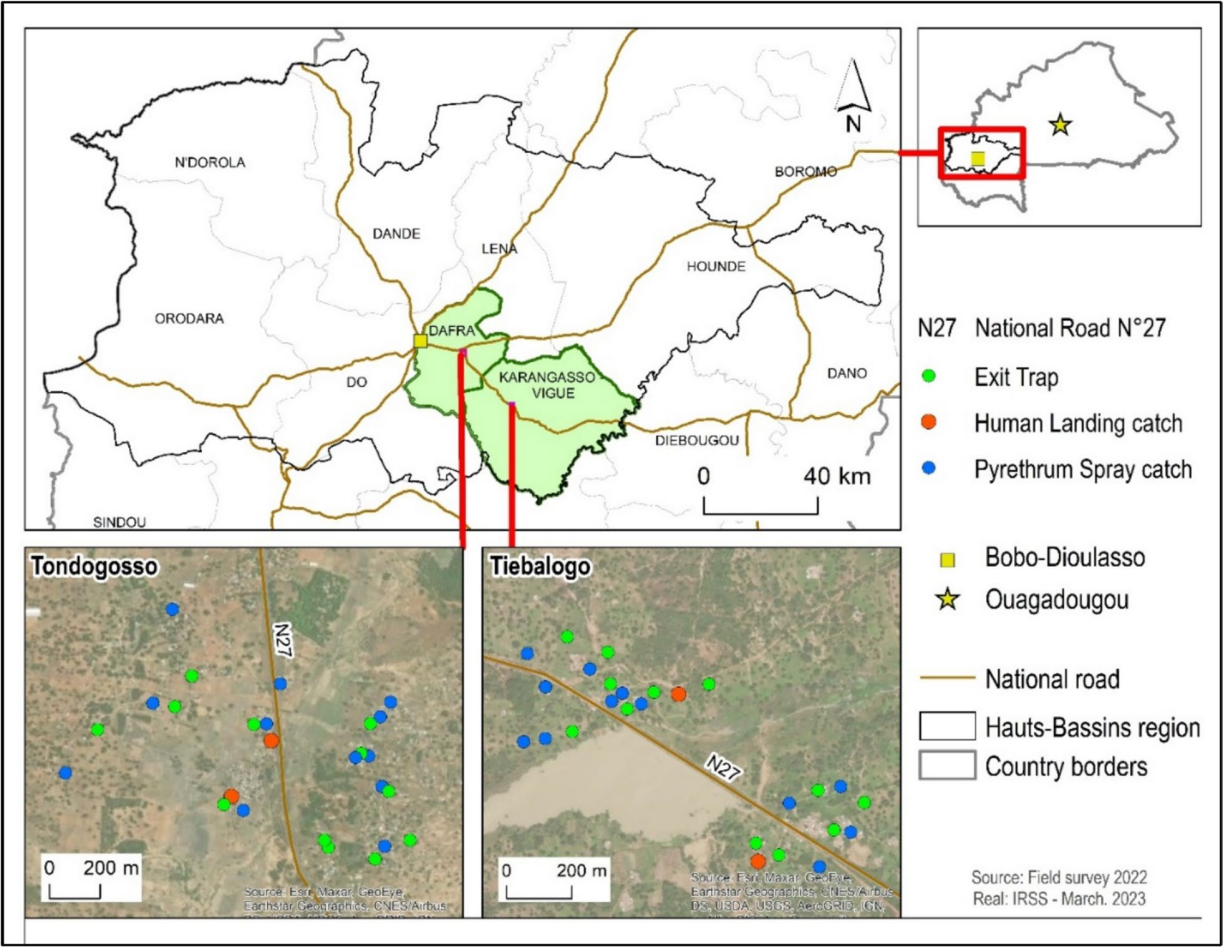
Distribution of trapping methods in households in the study sites

Two villages were selected for mosquitoes’ collection: Tiebalogo located in the Karangasso-Vigué implementation unit and Tondogosso (sentinel village) located in the Dafra implementation unit. Tiebalogo village was select in replacement of the sentinel village (Djosso) primarily selected for the study. But due to security issue during the study period Djosso was replace with the nearest village that presented less security issue. Both study sites are located between 30 and 55-km in the eastern part from Bobo-Dioulasso (Fig. 1).

### Study design

This study was a cross-sectional entomological survey conducted in two post-mass drug administration villages with population of fewer than 5,000 people. The villages were located in the Dafra and Karangasso-Vigué health districts. After more than ten years since the cessation of MDA, we used molecular xenomonitoring to determine whether there had been a resurgence of infection in the study communities. We collected mosquitoes, using three methods in each selected village, during the period of high mosquito abundance, e.i from August to December 2022.

### Collection and morphological identification of adult mosquitoes

The villages were divided into four zones and mosquitoes were sampled in each zone for two consecutive days per month. The sampling was done in selected houses according to WHO’s adult mosquitoes sampling methods [18]: Two for the Human Landing Collection (HLC) method (one indoor and one outdoor), 12 for the Pyrethrum Spray Collection (PSC) method and 12 for the Window Exit Trap (WET) method. Each collection site was georeferenced using a Garmin 62 handheld GPS device. For each household selected for the HLC method, volunteers were recruited to collect mosquitoes indoors and outdoors from 8:00 p.m to 6:00 a.m. Local volunteers and Institut de Recherche en Sciences de la Santé technicians performed PSC in the selected household between 6 a.m. and 8 a.m. Twelve WET were placed, one trap at the window of each selected house, and every morning between 6:00 to 8:00 a.m mosquitoes were collected in the trap using a mouth aspirator by technicians and local volunteers.

Mosquitoes were brought to the entomological laboratory of the Institut de Recherche en Sciences de la Santé, Direction Regional de l’Ouest. Once in the laboratory, mosquitoes were sorted into genera and identified morphologically using identification keys of Gilles and Coetzee [19]. Only *An. gambiae s.l*., *An. funestus*, and *An. nili* species were conserved in Eppendorff tubes under Silicagel grouped per species up to five mosquitoes by tube.

### Deoxyribonucleic acid (DNA) extraction and molecular detection of *Wuchereria bancrofti* in *Anophele*s species

Genomic DNA was extracted from anopheline species by using 2% Cetyl Trimethyl Ammonium Bromide (2% CTAB) as described in previous study [20]. We extracted genomic DNA from individually mosquitoes instead of pooling them for extraction. In the event of a positive pool, the individual DNA could be used to precise the specie of mosquito (*An. gambiae s.l*.) in the positive pool. Aliquots of 2 µL of DNA from the same species were grouped in pools of 1 to 10. The PCR assay described by Ramzy et al. [21] was used to detect *Wuchereria bancrofti*. The Specific repeated sequences SspI of *Wuchereria bancrofti* was amplified using two oligonucleotides’ primers (NV-1 and NV-2). In brief, the PCR was done in a final volume of 25 µL containing 2 µL of mosquito pool DNA, 0.25 µL of 10 µM NV-1) and 0.25 of 10 µM NV-2, 6.1 µL Firepol^®^ Master mix and 16.4 µL of molecular water. We used a positive control (*W. bancrofti*) extracted from *Anopheles* in our previous study [22] and a negative control (molecular water).

### Data analysis

Mosquito abundance was determined according to *Anopheles* species and collection method per site. The *Anopheles* species found in each village were pooled, and the percentage of infected pools for each species was calculated for each site by dividing the number of positive pools by the total number of pools for that species. We conducted data analysis using R version 4.4.1 software. The Chi-Square (χ2) test was used to compare the abundance of mosquitoes collected indoor and outdoor using HLC method in each village. A P value lower than 0.05 was considered statistically significant. The maps of the study area were drawn using Arc GIS, version 10.8.

## Results

### Abundance and composition of adult mosquitoes

A total of 2688 female mosquitoes were collected in both study sites (Tondogosso and Tiebalogo) between August and December 2022. The most predominant anopheline species was *An. gambiae s.l*. 2159 out of 2688 (80.3%). followed by *Culex* spp 412 (15.3%) *Mansonia* spp 20 (0.7%) and *Aedes* spp 3 (0.1%)1 (0.05%). Among the three mosquitoes’ collection methods employed, HLC has collected the highest number of mosquitoes 1802 (67%) followed by PSC method 647 (24.1%) and WET method 239 (8.9%).

**Fig. 2:**
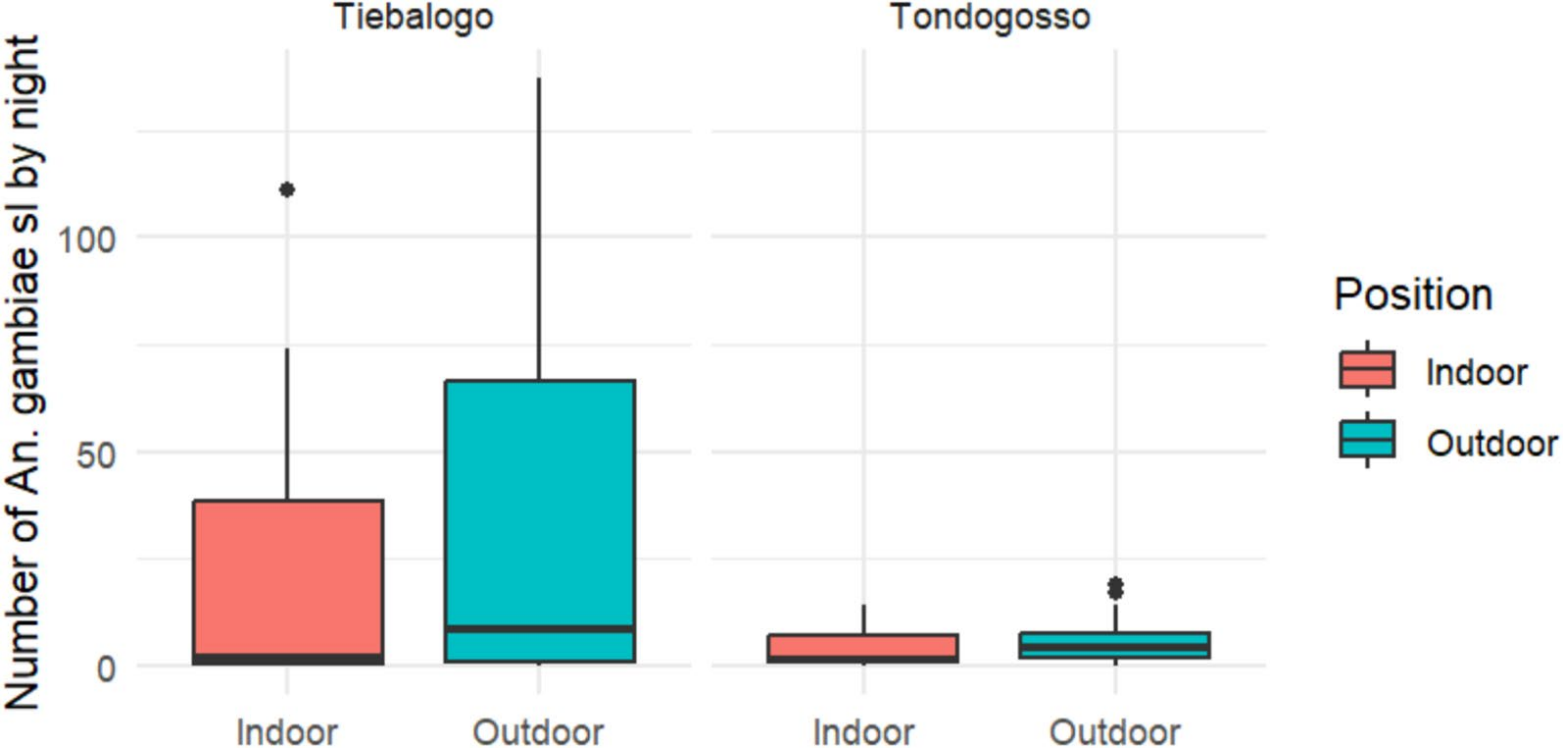
Mean number of *An. gambiae s.l.* collected by Human Landing Collection method per night Indoor and Outdoor in Tiebalogo and Tondogosso

In Tiebaologo, 2058 mosquitoes were collected, including 1786 (86.78%) of *An. gambiae s.l.* and in minor of *An. coustani*, *An. rufipes*, *An. nili* and *An. funestus*. The highest number of *An. gambiae s.l.* was obtained with HLC methods with more 1335 (74.7%) of total collection. The other major vector collected was *An. funestus* which represented 10 (0.49%) of the collection. Of the total *An. gambiae s.l.* collected by HLC, 822 (61.57%) were collected outdoor and 513 (38.43%) indoor. There was no significant difference between abondance of mosquito collected indoor and those collected outdoor for *An. gambiae s.l.* (χ2: p-value = 0.218) in Tiebalogo. In Tondogosso, 630 mosquitoes were collected, including 373 (59.21%) *An. gambiae s.l*.*,* 48 (7.62%) *An. rufipes*, and 2 (0.32%) *An. funestus* (Table 1). *An. gambiae s.l.* was the most frequent specie collected and by HLC in this site (Table 1). There was no statistical difference between the abundance of *An. gambiae s.l.* collected indoor and outdoor in Tondogosso (χ2: *p*-value = 0.279) (Fig. 2).

**Table 1:** Abundance and mosquitoes species composition by collection method in study sites

Culicidae species	Tondogosso				Tiebalogo				Total
	HLC	PSC	WET	Total (%)	HLC	PSC	WET	Total (%)	
*An. gambiae s.l*	234	85	54	373 (59.21)	1335	351	100	1786 (86.78)	2159 (80.32)
*An. funestus*	2	0	0	2 (0.32)	4	2	4	10 (0.49)	12 (0.45)
*An. nili*	0	0	0	0 (0)	10	0	1	11(0.53)	11 (0.41)
*An. rufipes*	36	7	5	48 (7.62)	0	16	5	21(1.02)	69(2.57)
*Aedes spp*	0	0	1	1(0.16)	1	1	0	2(0.10)	3(0.11)
*Culex spp*	95	62	47	204 (32.38)	65	121	22	208(10.11)	412 (15.33)
*An. coustani*	0	0	0	0 (0)	1	1	0	2 (0.10)	2 (0.07)
*Mansonia spp*	2	0	0	2 (0.32)	17	1	0	18(0.87)	20 (0.74)
Total	369	154	107	630	1433	493	132	2058	2688

### Detection of *Wuchereria bancrofti* DNA in *Anopheles* spp

A total of 2142 mosquitoes were analysed for *Wuchereria bancrofti* infection including 1767 from Tiebalogo and 375 mosquitoes from Tondogosso (Table 2). The number of pools per species in each village is described in Table 2. For both study sites, all pooled of *An. gambiae s.l.*, *An. funestus* and *An. nili* were negative for *Wuchereria bancrofti* DNA. There were no *Anopheles* spp infected with* Wuchereria bancrofti.*

**Table 2:** Percentage of infection and number of *Anopheles* species per pool by site

Study site	*An.* species	Number of mosquitoes	Number of pools	Number of positive pools	Percentage of infection
Tiebalogo	*An. gambiae s.l*	1746	175	0	0
	*An. funestus*	10	1	0	0
	*An. nili*	11	2	0	0
	Total	1767	178	0	0
Tondogosso	*An. gambiae s.l*	373	38	0	0
	*An. funestus*	2	1	0	0
	Total	375	39	0	0

## Discussion

The Hauts-Bassins Implementation Units ceased Mass Drug Administration in 2011, having met the TAS criteria set out by the WHO [23]. We carried out entomological survey during high abundance period of mosquitoes, employing molecular xenomonitoring as an additional tool to demonstrate that transmission had not resurged after 10 years of Stop MDA. No vectors were infected with *Wuchereria bancrofti*, in the study sites showing the absence of resurgence after ten years of MDA’s cessation. These results corroborated the findings of TAS which led to the halt of MDA in this region. This study is undoubtedly the first in Burkina Faso that provides additional evidence of LF elimination in a post-MDA area with another tool, apart from TAS.

MX in LF is more sensitive than the serological method used for TAS, because it detects parasite DNA within the human communities by analysing the major parasite vectors responsible for lymphatic filariasis. It also helps monitor settings progressing toward LF elimination [24–26]. Moreover, a recent study demonstrated a robust correlation between microfilaremia prevalence in humans and in major positive vectors using MX, in small area [27]. This provides additional evidence of the effectiveness of MX as a more sensitive tool for identifying residual infections in setting with low microfilarial prevalence. Regarding onchocerciasis surveillance, the WHO has recommended using MX in post-MDA area to demonstrate transmission interruption [28]. However, with regard to Lymphatic filariasis the WHO recommends using MX to support LF surveillance activities of the programmes but not to demonstrate transmission interruption because the standardization of mosquito collection method is still lacking [25]. The findings of our study may contribute to existing efforts to standardise the mosquito collection method for molecular xenomonitoring of lymphatic filariasis particularly for *Anopheles* mosquitoes [29].

Among the vector of LF in Western Africa, *An. gambiae s.l.* was the most frequently encountered vector of filariasis in our study sites. This vector is widely distributed throughout the country and is also the main vector of malaria [30]. In a previous study conducted in the South-west region of Burkina Faso, where lymphatic filariasis persists, *An. nili* that were collected were positive for *Wuchereria bancrofti*, justifying its inclusion in this study's MX process [32]. In both study sites, *An. nili* was only found in the village of Tiebalogo and in few numbers.

The most effective method for collecting the vectors was through HLC followed by PSC. HLC method has also been demonstrated to be the most effective technique for the collection of *An. gambiae s.l.* in previous studies on the entomological surveillance of lymphatic filariasis in Burkina Faso [32]. Our finding is different from those observed in Togo where PSC was the most effective method to collect *Anopheles* mosquitoes than HLC [24]. In Burkina Faso, HLC remains the optimal method for anopheline collection. Though HLC is an effective method in collecting *Anopheles* for MX, it presents an ethical challenge. Therefore, the sampling tools to be employed in replacement of the HLC method for lymphatic filariasis surveillance in the context of post-MDA remain topical in Burkina Faso and everywhere where the HLC has been the efficient method to sample LF vector over the years. This calls for the continuing research for adequate tools to sample *Anopheles* mosquitoes indoor and outdoor for LF molecular xenomonitoring in replacement of HLC.

The mass treatment campaigns conducted by the national control programme to eliminate lymphatic filariasis in Burkina Faso have resulted in the elimination of lymphatic filariasis in 61 out of 70 health districts in Burkina Faso. In these districts, mass treatments have been halted in accordance with the TAS criteria as defined by the WHO [31]. The implementation of molecular xenomonitoring as a monitoring tool in these districts would be a significant advantage for the surveillance of residual lymphatic filariasis transmission and progress toward elimination with the objective of providing evidence of interruption and ultimately preparing the dossier for elimination of lymphatic filariasis at the national level. In the meantime, the results of this study provide additional entomological evidence for the national neglected tropical disease control programme in its efforts to document the elimination of lymphatic filariasis 10 years after the cessation of MDA in the Hauts-Bassins region.

In addition to the existing serological tools for post-MDA surveillance, it would be interesting to add MX to the package of tools. It could be extended to other IU as a post-MDA additional surveillance tool to build evidence of elimination. It would be essential to determine a sufficient number of villages to be representative of the implementation unit.

## Conclusions

This study showed that none of the *Anopheles* mosquitoes screened were infected with *Wuchereria bancrofti* in either study village after 10 years of stopping MDA providing further evidence that there is no transmission occurring in the post-TAS area of Hauts-Bassins. The molecular xenomonitoring, which is used as a proxy method to detect *Wuchereria bancrofti* infection in humans could also be added as a complementary tool for monitoring transmission in post-MDA areas. This would provide evidence that interruption has been achieved after stopping MDA. These findings could help the national program for neglected tropical diseases to make appropriate decisions about where to extend molecular xenomonitoring.

## Data Availability

The datasets used to support these results are available from reasonable request to the corresponding author.

## Abbreviations

MDA: Mass Drug Administration
MX: Molecular Xenomonitoring
TAS: Transmission Assessment Survey
HLC: Human Landing Collection
WET: Window Exit Trap
PSC: Pyrethrum Spray Collection
LF: Lymphatic Filariasis
